# Water consumption and biomass production of protoplast fusion lines of poplar hybrids under drought stress

**DOI:** 10.3389/fpls.2015.00330

**Published:** 2015-05-19

**Authors:** Anne Hennig, Jörg R. G. Kleinschmit, Sebastian Schoneberg, Sonja Löffler, Alwin Janßen, Andrea Polle

**Affiliations:** ^1^Department for Forest Botany and Tree Physiology, Büsgen-Institute, Georg-August University of GöttingenGöttingen, Germany; ^2^Department Forest Genetic Resources, Northwest German Forest Research InstituteHann. Münden, Germany; ^3^Department Ecoinformatics, Biometrics and Forest Growth, Büsgen-Institute, Georg-August University of GöttingenGöttingen, Germany; ^4^Department for Monitoring and Forest Development, Forest Research Institute EberswaldeEberswalde, Germany

**Keywords:** polyploidy, *Populus*, abiotic stress, short rotation coppice, stomatal morphology, carbohydrate concentration

## Abstract

Woody crops such as poplars (*Populus*) can contribute to meet the increasing energy demand of a growing human population and can therefore enhance the security of energy supply. Using energy from biomass increases ecological sustainability as biomass is considered to play a pivotal role in abating climate change. Because areas for establishing poplar plantations are often confined to marginal sites drought tolerance is one important trait for poplar genotypes cultivated in short rotation coppice. We tested 9-month-old plants of four tetraploid *Populus tremula* (L.) × *P. tremuloides* (Michx.) lines that were generated by protoplast fusion and their diploid counterpart for water consumption and drought stress responses in a greenhouse experiment. The fusion lines showed equivalent or decreased height growth, stem biomass and total leaf area compared to the diploid line. The relative height increment of the fusion lines was not reduced compared to the diploid line when the plants were exposed to drought. The fusion lines were distinguished from the diploid counterpart by stomatal characteristics such as increased size and lower density. The changes in the stomatal apparatus did not affect the stomatal conductance. When exposed to drought the carbohydrate concentrations increased more strongly in the fusion lines than in the diploid line. Two fusion lines consumed significantly less water with regard to height growth, producing equivalent or increased relative stem biomass under drought compared to their diploid relative. Therefore, these tetraploid fusion lines are interesting candidates for short rotation biomass plantation on dry sites.

## Introduction

The world primary energy demand is increasing (Berndes et al., 2003; Asif and Muneer, 2007; Doman et al., 2014). About 11% of this demand was met by renewable sources as hydropower, biomass, biofuels, wind, geothermal and solar energy in 2008 (Chum et al., 2011). Biomass from bioenergy plants is expected to have a good potential to meet the increasing demand for global primary energy (Weih, 2004; Karp and Shield, 2008; Weih et al., 2014). Compared to fossil fuels, biomass contributes only marginally to the emission of greenhouse gasses (Weih, 2004), which reached their highest levels in history during the past decade and are main drivers of climate change (IPCC, 2014). Biomass is therefore considered to contribute substantially to the alleviation of climate change challenge (Weih, 2004; Karp and Shield, 2008). The establishment of short rotation coppice (SRC) is one possibility of generating biomass for energy purposes. SRC refers to plantations of fast growing trees and shrubs for biomass production with high initial growth and a rotation time of 3–5 years (Karp and Shield, 2008). The plantations of SRC show high biomass production and carbon dioxide fixation, subsequent use of wood chips has low carbon abatement costs (BMEL, 2007). In 2012, SRC covered an area of 5000–6000 ha in Germany, i.e., only 0.0003% of the managed agricultural land (von Wühlisch, 2012; BMEL, 2014) indicating a high potential to increase the area of SRC-cultivated land.

It has been suggested that conflicts in land use for food or biomass production can be diminished by establishing SRC on marginal sites, that are affected by pollution, salinization or low water and mineral supply (Kuzovkina and Quigley, 2005; Karp and Shield, 2008; Polle et al., 2013). Therefore, species and varieties with high drought tolerance are required (Karp and Shield, 2008; Weih et al., 2014). Some species of the genus *Populus* can meet the objective of low water demand, i.e., *Populus tremula* (L.). Their growth is more tolerant to drought than that of silver birch, Scots pine or Norway spruce (Jarvis and Jarvis, 1963). Both *P. tremula* and *P. tremuloides* (Michx.) are naturally occurring in areas with dry continental climate (Jarvis and Jarvis, 1963; Viereck et al., 1986). Thus, it can be assumed that hybrid aspen (*P. tremula* × *P. tremuloides*) are also drought tolerant. However, biomass yield highly depends on the availability of water and drought tolerance can also be achieved at the expense of biomass (Araus et al., 2002; Bogeat-Triboulot et al., 2006; Cattivelli et al., 2008). Poplars are needed that combine enhanced drought tolerance and reasonable biomass production. As drought periods are likely to increase with climate change (Regier et al., 2009) drought tolerance and the maintenance of growth are important breeding objectives (McKendry, 2002; Cattivelli et al., 2008). Another important trait for poplar cultivated in SRC is the propagation via stem cuttings as this considerably reduces the investment costs (Stanturf et al., 2001). Poplar species of the section Tacamahaca and Aigeros, for example, *P. nigra* and *P. trichocarpa* × *P. deltoides* show this specific trait in contrast to species of the section Populus (Stanturf et al., 2001). To combine the drought tolerance of *P. tremula* × *P. tremuloides* and the ability of propagation via stem cuttings of the species *P. nigra* and *P. trichocarpa* × *P. deltoides* somatic hybridization via protoplast fusion appears to be a promising approach.

Genome duplication (polyploidization) has naturally occurred in the evolution of several plant species including crops like *Gossypium hirsutum, Triticum aestivum*, and *Oryza sativa* but also in tree species such as *Populus* (Finnegan, 2002; Blanc and Wolfe, 2004; Rausher, 2007). Duplicated genes typically show a diversification in functions or subfunctionalization (Adams and Wendel, 2005). Several studies revealed that gene doubling influenced transcription levels by epigenetic alterations in the cytosine methylation or silencing of the ribosomal RNA resulting in a variation of morphology and phenotype (Finnegan, 2002; Liu and Wendel, 2003; Adams and Wendel, 2005). Silencing of polyploid genes can be organ-specific and was observed to occur even reciprocally (Adams et al., 2003).

Octaploid tobacco plants showed increased survival times over their tetraploid counterparts when exposed to stresses like cold, shade, water logging, nutrient deficiency and drought (Deng et al., 2012). Decreased susceptibility of polyploid varieties to drought was detected in crop species (*Triticum*), herbaceous species (*Lonicera, Spathiphyllum, Nicotiana*) as well as in tree species (*Betula*) (Li et al., 1996, 2009; Xiong et al., 2006; van Laere et al., 2010; Deng et al., 2012). Polyploidy can induce morphological changes in leaf characteristics that are associated with drought tolerance like an increased leaf thickness, a smaller total leaf area and an enhanced leaf mass per area (Kubiske and Abrams, 1992; Li et al., 2009). For instance, greater stomatal length and reductions in stomatal density, that are characteristics of plants in xeric habitats, were observed in polyploid *Betula* and *Spathiphyllum* (Abrams et al., 1994; Li et al., 1996; van Laere et al., 2010). Polyploidy can also influence the metabolic performance resulting in an induction of superoxide dismutase and catalase and consequently decreased accumulation of reactive oxygen species (ROS) (Deng et al., 2012). In *Lonicera*, photosynthesis, pre-dawn leaf water potential and stomatal conductance were less affected in drought-treated tetraploid variants compared to their diploid relatives (Li et al., 2009).

Plants cope with stresses like drought either by stress avoidance or stress tolerance where stress avoidance is referred to as the plants ability to minimize the adverse effect and tolerance as the capacity to endure unfavorable conditions (Puijalon et al., 2011). For example, osmotic adjustment with inorganic ions, carbohydrates and organic acids or changes in tissue elasticity can enhance plant's drought tolerance (Touchette et al., 2009). Osmotic adjustment plays a role in minimizing yield loss when drought occurs (Cattivelli et al., 2008). Therefore, analysis of osmotic adjustment under different drought conditions has been suggested as an effective selection criterion for drought tolerant genotypes (Cattivelli et al., 2008). Drought avoidance occurs when plants reduce their transpiration surface by leaf shedding for example (Gaur et al., 2008; Fischer and Polle, 2010). When facing water limiting conditions both drought tolerance and avoidance mechanisms are often combined to enhance physiological adjustments in plants (Touchette et al., 2009).

Because of the importance of poplars as bioenergy crops, the overarching goal of this study was to test polyploid species obtained by protoplast fusion for their performance under drought Here, diploid plant material of hybrid aspen (*P. tremula* × *P. tremuloides*), black poplar (*P. nigra* L.) and cottonwood hybrids (*P. trichocarpa* Torr. and Gray × *P. deltoides* Bartram ex Marsh.) were used to generate polyploid lines by protoplast fusion. We obtained four tetraploid lines of *P. tremula* × *P. tremuloides* and compared them to their diploid counterpart. To examine the suitability of the diploid and protoplast fusion lines for cultivation on dry sites, we investigated (i) morphological and physiological effects of polyploidization, (ii) water consumption, and (iii) drought stress performance in a greenhouse experiment.

## Materials and methods

### Plant material

Five-year-old trees of *P. tremula* × *P. tremuloides* (“Münden 2”), 20-old trees of *P. nigra* and 25-year-old trees of *P. trichocarpa* × *P. deltoides* were used for establishing *in vitro* cultures. Terminal and axillary winter buds of 1-year-old shoots were harvested and processed after a protocol modified according to Ahuja (1984). Buds were washed in tap water and sterilized in 70% ethanol (Carl Roth GmbH and Co. KG, Karlsruhe, Germany) with 0.1% L-ascorbic acid (Sigma Aldrich Laborchemikalien GmbH, Hannover, Germany) for 20 s and in sodium hypochlorite (Carl Roth GmbH and Co. KG, Karlsruhe, Germany) (supplemented with 2 drops of Tween 20 (Carl Roth GmbH and Co. KG, Karlsruhe, Germany) for 20 min. Material was then washed three times for 5 min in sterilized tap water. After shoot development plantlets were subcultured every 4 weeks on MS-Medium (Murashige and Skoog, 1962) supplemented with 0.2 ppm 6-benzylaminopurine (Fluka Chemie GmbH, Steinheim, Germany), 2% sucrose (Carl Roth GmbH and Co. KG, Karlsruhe, Germany) and 2.9 g/L Gelrite (Duchefa Biochemie, Haarlem, Netherlands). Protoplast fusion of the *in vitro* poplar clones was established according to modified protocols of Sasamoto et al. (2006) and Guo and Deng (1998) by the company Phytowelt GreenTechnologies GmbH as described previously (Lührs et al., 2010, 2012; Efremova et al., 2013). Shoot cultures from fusion products were regenerated as separate lines. Three lines of three protoplast fusion experiments between *P. tremula* × *P. tremuloides* (“Münden 2”) and *P. trichocarpa* × *P*. *deltoides* (B19) and one line out of one protoplast fusion between *P. tremula* × *P*. *tremuloides* (“Münden 2”) and *P. nigra* were used. The lines were micropropagated and rooted *ex vitro* by directly transferring them to substrate (nursery substrate (N: 250 mg/L P: 140 mg/L, K: 250 mg/L), Kleeschulte Erden GmbH, Rüthen, Germany) under a foil tunnel equipped with a fog system. Plantlets were hardened by reducing air humidity gradually during 4 weeks. Rooted plants were transferred into 1.3-L pots on October 2012 (substrate composition as above blended with one gram long-term fertilizer Osmocote Exact lo start 8–9 M (1 g/L, N:P:K = 15:8:10 + 3 MgO), The Scotts Company LLC, Heerlen, Netherlands per liter soil) and cultured in the greenhouse. For hibernation temperature was decreased according to ambient conditions but did not drop below 5°C. In April 2013, plants were transferred to three-liter pots (substrate composition above) with long-term fertilizer (1 g/L, Osmocote Exact Standard 5–6 M (1 g/L, N:P:K = 15:9:12 + 2 MgO), The Scotts Company LLC, Heerlen, Netherlands). Plants were cultured in the greenhouse and watered to pot capacity until the start of the drought treatment on July 11th, 2013, when the plants were 9 month old.

### Experimental design

**Figure 1:**
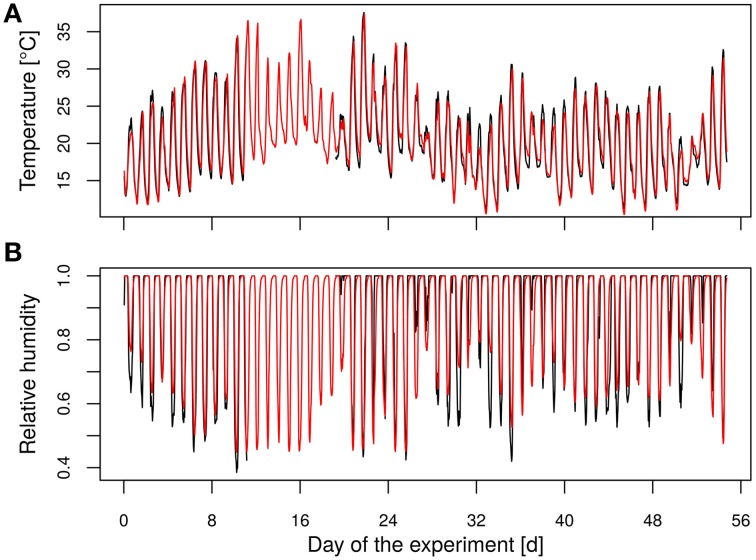
**Time course of (A) the temperature and (B) the relative humidity**. (Black, measured data; red, modeled data).

Four lines from protoplast fusion and the original diploid hybrid aspen (“Münden 2”) were used in a greenhouse experiment. Ten plants of each clone were randomly chosen as control and ten as treatment plants. Two plants each of four lines, still planted in three-liter pots, were placed into one box (eight plants) according to a scheme applying maximal space to the plants of one line. The plants' positions were changed in each box in order to let all lines pass all positions of the boxes (four different distributions, one was repeated). In total, 12 lines (240 plants) were tested, but we focus in this analysis on the four fusion lines of the phenotype *P. tremula* × *P. tremuloides*. During the experiment the boxes were rotated daily to avoid position effects. No artificial light was supplemented. Temperature and relative air humidity during experimental time ranged from 16°C to 37°C/37% to 60% (day) and from 11°C to 15°C/80% to 99% (night), respectively (Figures 1A,B). Because of data logger failure inside the greenhouse during 8 days (day 11 to day 19), we used hourly data of existing data pairs for inner and outer temperature to generate a linear regression model (Equation 1) with the outer temperature (*x*_1_) as predictor variable.

(1) $$y=\beta_{0}+x_{1}\;\beta_{1}+\varepsilon$$

The modeled and measured temperatures showed a high correlation (*R*^2^ = 0.92, Figure 1A). The relative humidity was modeled using a generalized linear regression approach as the target variable ranged between 0 and 100%. Therefore, a logit function was applied (Equation 2) with the predicted temperature inside the greenhouse (*x*_1_) and the hour of the day (*x*_2_) as predictor variables.

(2) $$\hat{y}=\frac{\exp(\beta_{0}+\beta_{1}x_{1}+\beta_{2}x_{2})}{1+\exp\left(\beta_{0}+\beta_{1}x_{1}+\beta_{2}x_{2}\right)}$$

For more flexibility of the model (Equation 2) was extended by flexible splines according to Wood (2011). The predicted values of the relative humidity were highly correlated with the measured values (*R*^2^ = 0.79, Figure 1B). The vapor pressure deficit (vpd) was determined using the predicted temperature and relative humidity data with the Clausius-Clapeyron relationship according to Hartmann (1994).

All plants were watered twice up to saturation before starting the experiment. Then, control plants were watered daily to pot capacity and treatment plants were gradually dried until a soil water content of 10 vol.-% was reached and kept at the 10 vol.-%-level for 1 week. To enable uniform drying of plants within one line volumetric soil water content was measured daily using a soil moisture sensor (TRIME PICO32; Imko GmbH, Ettlingen, Germany). Two measurements were performed per pot and the mean soil moisture per line was calculated for all plants per line. Plants, whose soil moisture dropped below the average level of their line were watered to reach the mean volumetric soil moisture level. This procedure ensured that all plants of one line reached the 10 vol.-% soil moisture target level at the same time point. The 10 vol.-% periods for the different lines were staggered and first occurred in the diploid line at day six after starting the experiment and appeared last for fusion line 27-09 at day 47 (**Figure 4A**). After culturing the plants at this level for 1 week, treatment plants were not watered anymore but still investigated. The experiment ended at day 56, when all plants were harvested for biomass analysis.

### Morphology and basic characterization

#### Leaf and stomatal morphology

Leaf morphology of *in vitro* plants of the fusion lines and the original diploid clones were compared and the fusion lines were associated to one of the original clones phenotypically. The stomatal density and stomatal size were analyzed by leaf impressions from abaxial and adaxial leaf surfaces. Impressions were taken on the fifth fully expanded leaf of five plants per line before starting the experiment using clear nail polish. The stomatal density was determined by counting the stomata on three randomly chosen sections (0.303 mm ^*^ 0.303 mm) of every preparation using a microscope (400 X magnification, Zeiss Axio Observer Z1, Zeiss, Oberkochen, Germany). Subsequently, the number of stomata per square millimeter was calculated. For determining the stomatal size, length of three guard cells was measured in each section. The stomatal area index (SAI) was calculated as a mean of stomatal size of three stomata of each leaf section on abaxial surface and multiplied with the stomatal density of this section (Ashton and Berlyn, 1994).

#### Height and stem biomass

The height of all plants was measured at the beginning of the experiment (H_d0). For basic characterization height growth is shown for the data of the control group before starting the experiment (H_d0).At the end of the experiment all plants were separated into stem and root segments and dried at 103°C for 5 days. Dry weight of the stem (DW_end) was determined without the leaves. Stem biomass is displayed for the data of the control group.

#### Total leaf area and leaf mass per area

For determining total leaf area and calculating a ratio between leaf mass and total leaf area all leaves of six control plants from each line were harvested, weighed (Sartorius Basic BA 210, Sartorius Weighing Technology GmbH, Göttingen, Germany) and scanned with a standard scanner at the end of the experiment. Leaf area was calculated from the scans using the program Histo Version 1.0.1.2. (Datinf, Tübingen, Germany).

### Genetic analyses

#### Ploidy level

Relative DNA content was analyzed by flow cytometry from leaves of *in vitro* cultures (Plant Cytometry Services, Netherlands).

#### DNA extraction

The protocol of Dumolin et al. (1995) was used for total DNA isolation from leaves of *in vitro* cultures. Differing from the protocol the pellet was eluted in 75 μl 1 × TE Rnase.

#### nSSR analysis

Eleven primers (ORPM0023, ORPM1031, ORPM1249, ORPM1261, PMGC0433, PMGC2163, WPMS05, WPMS09, WPMS12, WPMS14, GCPM2768) that were located on nine linkage groups were selected for analysis of nuclear simple sequence repeats (nSSR; web.ornl.gov/sci/ipgc/ssr_resources.htm; van der Schoot et al., 2000; Smulders et al., 2001; Tuskan et al., 2004). Polymerase chain reaction was performed as described by van der Schoot et al. (2000), Smulders et al. (2001), Eusemann et al. (2009). nSSR fragment length analysis was carried out using a LI-COR sequencer (4300 DNA analyzer, LI-COR Biosciences, Bad Homburg, Germany). For genotype analysis the software Saga v3.0 (LI-COR Biosciences, Bad Homburg, Germany) was used.

### Drought-related parameters

#### Water consumption and plant vitality

All water volumes that were added to the treatment plants within the 10 vol.-% soil moisture level for the 7 day period were recorded. The wilting and the desiccation of the leaves were determined for plant vitality analysis for treatment and control plants. A leaf was regarded as wilted, when its blade was in a parallel position to the shoot, i.e., the angle between shoot and leaf blade was 0°–10°. Leaves that showed an angle of more than 10° were considered as not wilted. Desiccation was observed as the percentage of dried leaves. Both parameters were observed daily during the drought period for all plants and were classified into five categories (0, 25, 50, 75, 100%) according to Table 1. Then, a mean was calculated for each line and normalized to the last irrigation event.

**Table 1:** **Classification of vitality**.

**Scale**	**Wilting percentage of wilted leaves [%]**	**Desiccation percentage of dried leaves [%]**
0	0	0
1	25	25
2	50	50
3	75	75
4	100	100

#### Stomatal conductance and carbohydrate concentration

The stomatal conductance was determined after 7 days exposure to 10 vol.-% soil moisture with a porometer (AP4, Delta-T Devices, Cambridge, Great Britain) on the eighth fully expanded leaf of control and treatment plants in the morning between 8:30 h and 11:30 h. That was on the following days of the experiment: 27-01: day 13, 27-09, day 54, 27-10: day 18, 27-11: day 15, 27-12: day 15. For analysis of carbohydrate concentration leaf samples were taken from the upper fourth fully expanded leaf in five repetitions for control and treatment plants. Sampling was conducted after 7 days of exposure to 10 vol.-% soil moisture level during the morning (10–12 h). The material was immediately stored at −20°C until further preparation. For total soluble carbohydrates the protocol of Yemm and Willis (1954) was modified as follows: Samples were extracted in 50% ethanol, incubated with anthrone for 10 min at 98°C and afterwards immediately cooled down in iced water. Absorbance was then measured at 620 nm against a blank that included pure methanol instead of the methanol-extracted sample.

#### Relative height and stem increment

The height of all plants was measured for control and treatment plants after culturing at 10 vol.-% soil moisture (H_10%) for 7 days and final height was observed at the end of the experiment (H_end). Relative height increment was calculated by dividing height increment (H_10% - H_d0) by initial height (H_d0). As the period until the 10 vol.-% soil moisture level was reached differed for the tested lines, the height increment was divided by the number of cultured (≡ days when plants were irrigated) days, i.e., for the lines 27-01, 27-09. 27-10, 27-11, and 27-12 as follows: 13, 54, 18, 22, 15. Relative stem increment was calculated for control and treatment plants as follows: First, a model between final stem dry mass (DW_end) and final height (H_end) was calculated using a logarithmic function. Then, the model was applied to initial height (H_d0) estimating initial stem biomass (DW_d0). Finally, the stem increment was calculated (DW_end—DW_d0), divided by the initial stem biomass (DW_d0) and then normalized to the number of days when plants were irrigated.

### Statistical analysis

All data were analyzed using the statistical software R (R Core Team, 2013). The parameters height, stem biomass, total leaf area, leaf mass per area, stomatal length and density, the stomatal area index and the vpd during the 10 vol.-% soil moisture periods were analyzed by an One-Way analysis of variance (ANOVA, Supplementary Table 1). The hypothesis H_0_ describing that no differences between the genotypes exist was rejected if *p* < 0.05. A *post-hoc* test (TukeyHSD) was used for determining significant differences between the genotypes (Supplementary Table 2). Two-Way ANOVA with independent factors genotype and treatment was conducted to test genotype, treatment and interaction effects on carbohydrate concentration, relative height increment and relative stem increment (Supplementary Table 1). For stomatal conductance the factor light was tested in addition (Supplementary Table 1). Using a *post-hoc* test (TukeyHSD) the dataset of control and treatment were analyzed together for determining significant differences for the parameters carbohydrate concentration, relative height and stem increment (Supplementary Table 2). For stomatal conductance the control and treatment were analyzed separately (Supplementary Table 2). Leaf wilting and desiccation was analyzed using a Wilcoxon signed-rank test. Here, a pairwise comparison was conducted for the genotypes (Supplementary Table 3). Differences between the lines were considered to be significant when *p* < 0.05. For determining the water consumption at 10 vol.-% soil moisture we calculated the sum of added water volumes per plant and week. The linear models according to Equations (3) and (4) were tested.

(3) $$y=\beta_{0}+x_{1}\beta_{1}+\varepsilon$$

(4) $$y=\beta_{0}+x_{1}\beta_{1}+x_{2}\beta_{2}+\varepsilon$$

where *x*_1_ indicates the genotype and *x*_2_ the stem height.

When significant differences between the models were found the one with the lowest residual sums of squares was applied. For testing if a model with enhanced parameters is equal to a model with reduced parameters (*H*_0_) the test statistic is calculated by Equation (5):

(5) $$F=\frac{\frac{1}{p-k}(SS_{reduced}-SS_{full})}{\frac{1}{n-p-1}SS_{full}}$$

with *p* number of parameter of the full model, *k* number of parameters of the reduced model and *n* number of observations. Equation (4) fitted the data better than Equation (3) (Supplementary Table 4). Differences among the genotypes were detected using Equation (4) by estimating the shift of the intercepts from a reference. Afterwards, this shift was tested for being equal to zero (H_0_) using the exact *F*-Test. H_0_ was rejected if *p* < 0.05. Each genotype served as reference using the R function “lm.” Thereby, all treatments were tested against each other.

## Results

### Morphological and genetic characterization

We investigated four hybrid aspen lines that were obtained after three protoplast fusion experiments between *P. tremula* × *P. tremuloides* and *P. trichocarpa* × *P. deltoides* and one protoplast fusion experiment between *P. tremula* × *P. tremuloides* and *P. nigra* in comparison to the original diploid clone of *P. tremula* × *P. tremuloides*.

**Figure 2:**
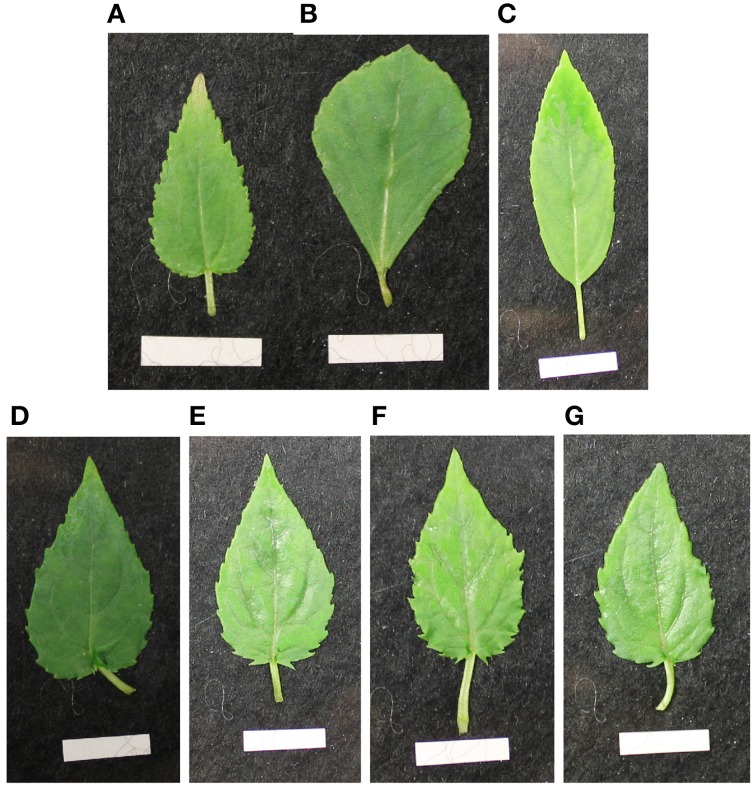
**Leaf morphology of *in vitro* leaves of the diploid original clones (A) *P. tremula* × *P. tremuloides* (27-01), (B) *P. nigra*, (C) *P. trichocarpa* × *P. deltoides* and the fusion lines (D) 27-09, (E) 27-10, (F) 27-11, (G) 27-12**. (The white scale bar at the bottom of the pictures measures 1 cm in length. Plants were subcultured for 4 weeks).

In the tested putative heterofusion lines no DNA from *P. nigra* or *P. trichocarpa* × *P*. *deltoides* was detected by nSSR analysis using 11 nSSR markers that were located on nine linkage groups (Table 2). This result suggested that only DNA of *P. tremula* × *P. tremuloides* was present. To get further insight into the genetic composition of the hybrid aspen lines flow cytometry was conducted. According to this analysis, the hybrid aspen lines showed a tetraploid set of chromosomes (Table 2). Our analyses, therefore, support that homofusion lines of hybrid aspen (*P. tremula* × *P. tremuloides*) were obtained, but no heterofusion lines with other poplar genotypes. This finding was also supported by the leaf morphology of the fusion lines that exhibited the *P. tremula* × *P. tremuloides* phenotype (Figure 2). In the following text, the putative hybrid aspen clones that originated from the protoplast fusion experiments are therefore named fusion lines. The original diploid *P. tremula* × *P. tremuloides* clone is referred to as the diploid line.

**Table 2:** **Morphological and genetic characterization of the diploid and the tetraploid hybrid aspen lines under optimal water supply (stem height was measured at the beginning of the experiment after 9 month in soil, other parameters were measured at the end of the experiment**.

**Line**	**Fusion partners**	**Leaf morphology**	Stem height [m ^*^ 10^−2^]	**Stem biomass [g plant^−1^]**	Total leaf area [m^2^^*^ 10^−2^ plant^−1^]	Leaf mass per area [g m^−2^^*^ 10]	**Ploidy level**	**nSSR**
27-01	–	P3	105.1 ± 17.6^c^	17.5 ± 8.3^b^	30.4 ± 7.7^b,c^	1.38 ± 0.15^a^	2n	P3
27-09	P3 × P9	P3	39.6 ± 17.3^a^	1.9 ± 1.2^a^	6.5 ± 1.7^a^	2.26 ± 1.98^c^	4n	P3
27-10	P3 × P9	P3	80.3 ± 16.7^b^	16.3 ± 10.0^b^	23.9 ± 12.7^b,c^	1.59 ± 0.23^b^	4n	P3
27-11	P3 × P9	P3	49.8 ± 11.8^a^	4.9 ± 3.5^a^	15.4 ± 7.8^a, b^	1.53 ± 0.26^a,b^	4n	P3
27-12	P3 × P7	P3	92.8 ± 18.4^b, c^	22.1 ± 7.3^b^	34.2 ± 6.0^c^	1.69 ± 0.21^b^	4n	P3

*For analyzing ploidy level and nSSR in vitro material was used. Data are means ± SE, n = 10 plants (stem height and stem biomass), n = 6 plants (total leaf area and leaf mass per area), superscripted letters indicate differences between the lines (Tukey HSD test, p < 0.05), P3: P. tremula × P. tremuloides, P7: P.nigra, P9: P. trichocarpa × P. deltoides)*.

For basic characterization of the fusion lines height at the beginning of the experiment after 9 month of soil culture and total leaf area, leaf mass per area and stem biomass production at the end of the experiment were compared to the diploid line under optimal water supply (Table 2). Plants heights were lower for the fusion lines 27-09, 27-10, and 27-11 than for the diploid line. Stem biomass of the fusion lines 27-09 and 27-11 was reduced. Furthermore, for the fusion line 27-09 the total leaf area was decreased compared to the diploid line. The leaf mass per area was increased in the fusion lines except for 27-11 suggesting enhanced leaf thickness for the fusion lines.

**Figure 3:**
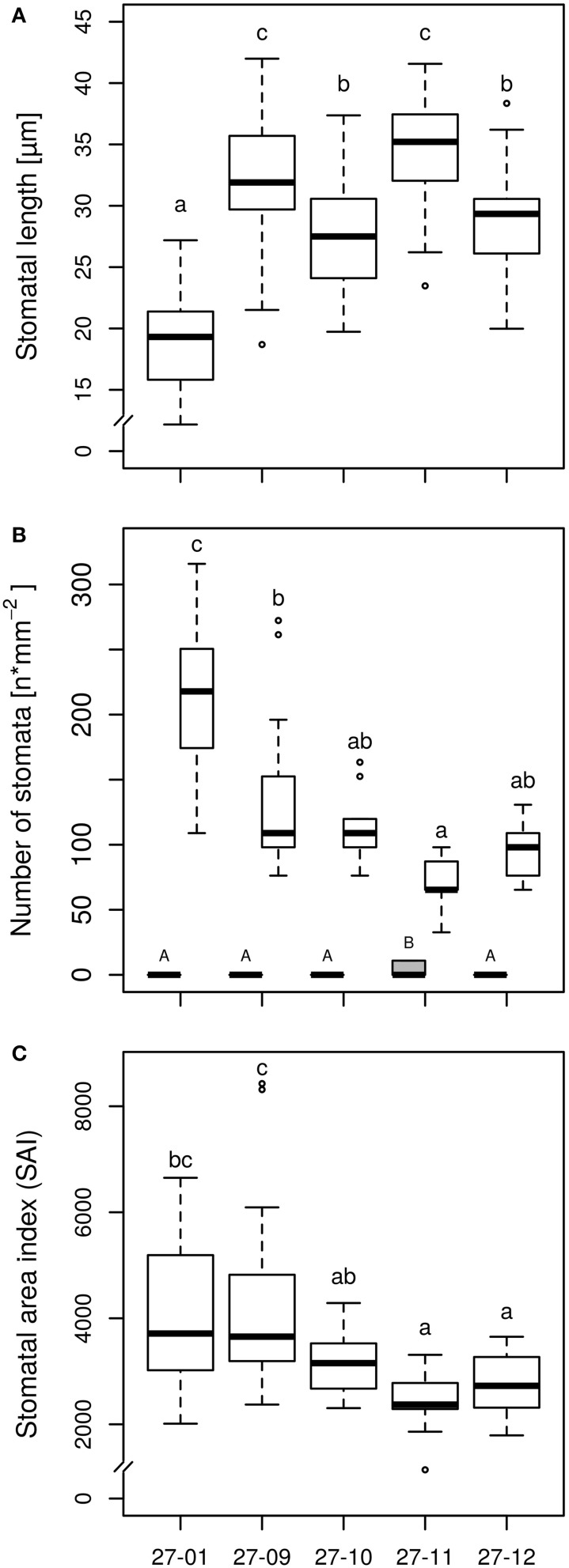
**(A)** Stomatal length on abaxial surface, **(B)** Stomatal density on adaxial and abaxial surface, and **(C)** Stomatal area index (SAI) on abaxial surface [samples were taken of the upper fifth fully expanded leaf of the diploid line and the tetraploid fusion lines before starting the experiment, stomatal length: *n* = 45 stomata per line, stomatal density: *n* = 15 leaf sections per line and leaf side (gray box, adaxial side; white boxes, abaxial side), SAI: mean stomatal length of three stomata of one leaf section multiplied by stomatal density of this leaf section, *n* = 15 leaf sections per line; 27-01: diploid, other lines: tetraploid. Boxplot definition: Outer transversal bars of the boxplots include 95% of the data, 50% of the data are defined by the boxes themselves, the median is shown by the highlighted line within the box. Outliers are represented by open circles. Different letters at the top of the boxes indicate significant differences between the lines (TukeyHSD test, *p* < 0.05). Capitals are used for the adaxial side, lowercase letters for the abaxial side].

Microscopy of leaf impressions that were taken of the abaxial leaf surface before starting the experiment revealed that the stomatal lengths were higher but the stomatal densities were reduced for all fusion lines in comparison to the diploid line (Figures 3A,B). Among the fusion lines stomatal lengths and densities differed. Fusion line 27-11, which showed the lowest stomatal density on lower leaf surface, exhibited stomata on the leaf adaxial surface. But the stomatal density on the adaxial leaf surface was only 10 stomata mm^−2^ compared to 75 stomata mm^−2^ on the abaxial leaf side, thus was significantly lower. None of the other lines, including the diploid line, developed stomata on the adaxial leaf surface. The stomatal area index was reduced for the fusion lines 27-11 and 27-12 compared to the diploid line (Figure 3C).

### Performance of the diploid and the fusion lines under drought

#### Water consumption and leaf vitality

**Figure 4:**
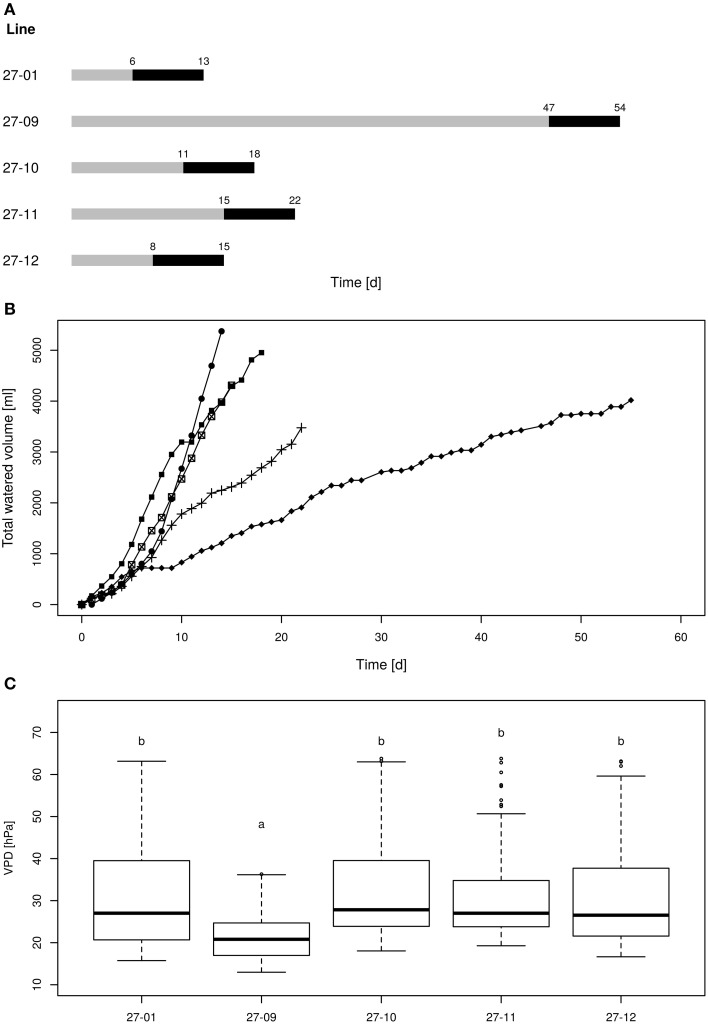
**(A)** Scheme of the drought periods, **(B)** cumulative water volumes applied to each line, **(C)** vapor pressure deficit during the 10 vol.-% levels [gray line: drying period until the 10 vol.-% soil moisture level was reached for all plants of one line, black line: 10 vol.-% soil moisture period; 27-01: circles, 27-10: squares, 27-11: crosses, 27-12: crossed squares. Boxplots are defined as explained in Figure 3. Different letters at the top of the boxes indicate significant differences between the lines (TukeyHSD test, *p* < 0.05)].

**Figure 5:**
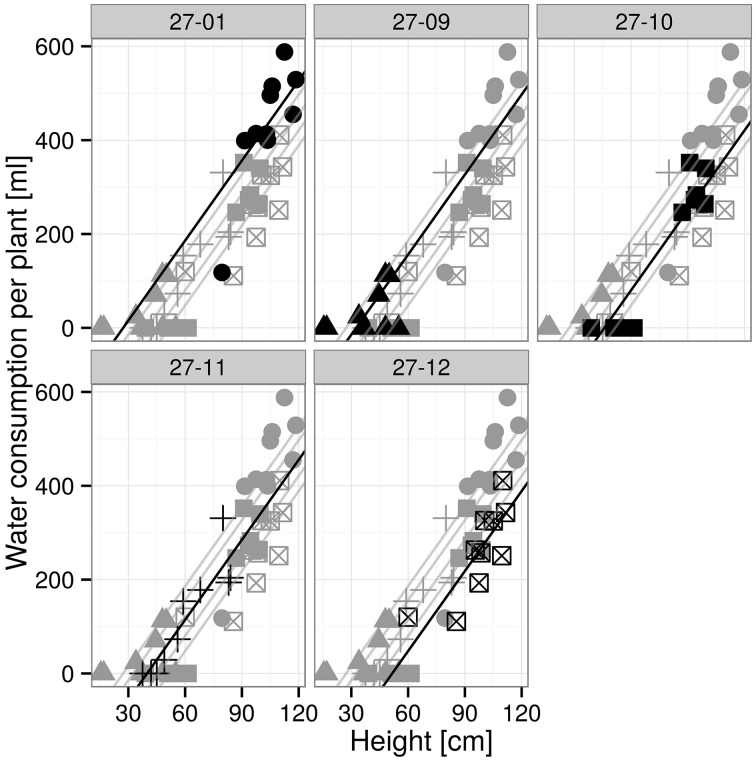
**Water consumption for 7 days at 10 vol.-% soil moisture level depending on height**. [*n* = 10 plants, data and the regression line of the respective plot are in black, others are visualized in gray; groups with different letters indicate significant differences between the lines (exact *F*-Test, *p* < 0.05): 27-01^a^, 27-09^a^, 27-10^b^, 27-11^ab^, 27-12^b^ (Supplementary Table 5)].

The plants were exposed to drought by gradually drying until soil moisture levels of 10 vol-% were reached (Figure 4A). Each line attained the target soil moisture at a different time point and was kept at this level for a period of 7 days (Figure 4A). Subsequently, the lines were not watered anymore. The water consumption was determined during the 7-day period at the 10 vol.-% soil moisture level. The diploid consumed more water than the fusion lines (Figure 4B). This was not only observed for fusion lines that were smaller than the diploid line, but was also detected for the fusion lines 27-10 and 27-12 when height was respected (Table 2, Figures 4B, 5). A further factor that may influence water consumption is a variation in the vpd. Although the periods when the plants experienced 10 vol-% soil moisture occurred at different time points, vpd levels did not differ except for the fusion line 27-09 (Figure 4C). Here, the vpd was lower compared to all other lines. Relative to the height this line consumed as much water as the diploid line and even more than the fusion lines 27-10 and 27-12 (Figure 5) that experienced higher vpd. Therefore, variation in vpd among the lines was not the reason for differences in the water consumption.

The linear regression analysis showed that the fusion lines 27-10 and 27-12 consumed significantly less water with regard to height than the diploid line (Figure 5).

**Figure 6:**
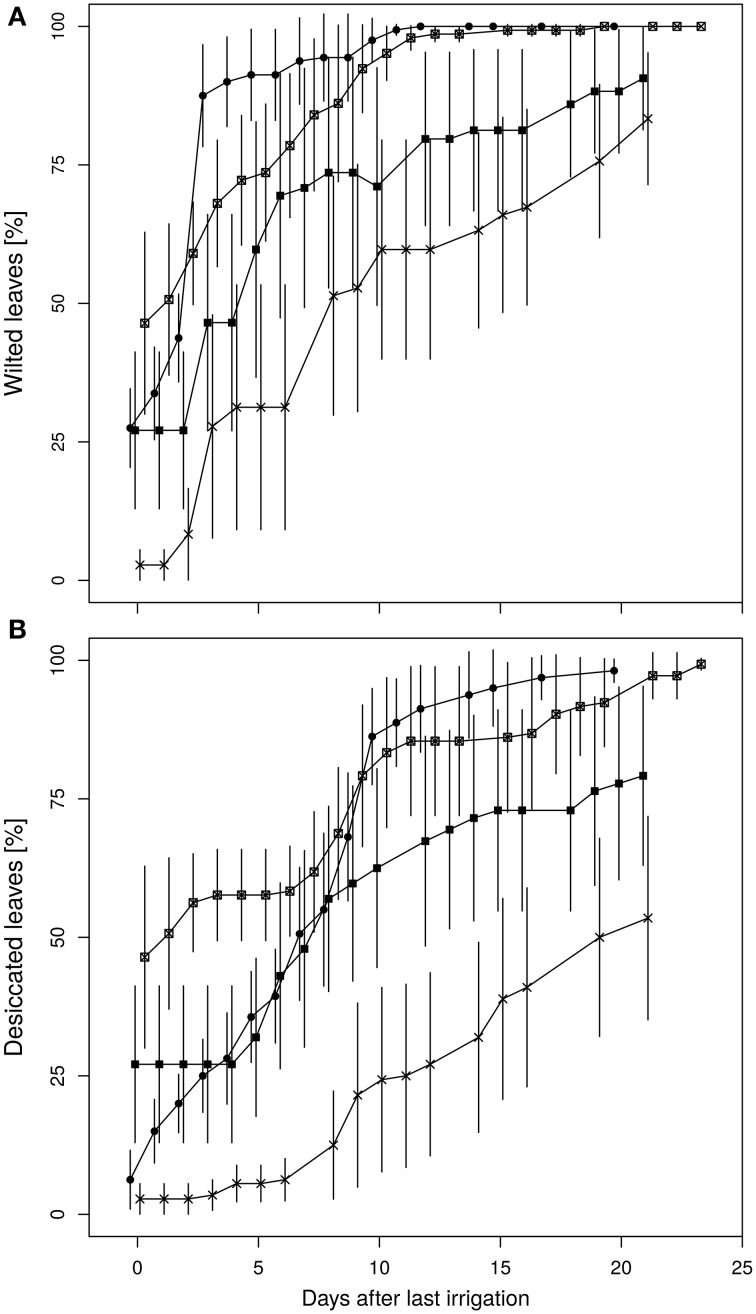
**Time course of (A) leaf wilting, (B) desiccation of leaves**. (Data are normalized to last irrigation event and are means ± standard errors, *n* = 10 plants. 27-01: circles, 27-10: squares, 27-11: crosses, 27-12: crossed squares).

Leaf wilting and desiccation were determined as indicators for plant vitality (Figures 6A,B). The wilting process of the diploid plants occurred more rapidly with a steeper increment than in the fusion lines. Average leaf wilting of about 85% already appeared at the third day after the final irrigation of the diploid line. Similarly strong wilting occurred in the fusion line 27-12 at day nine after the last watering, whereas this threshold was not observed for the fusion lines 27-10 and 27-11 during the whole experiment. Instead, desiccation of leaves was first visible in two fusion lines. The lines 27-10 and 27-12 showed a proportional leaf wilting of about 25 and 50%, respectively, already at the end of the 10 vol.-% soil moisture phase. Early leaf desiccation might have had an influence on leaf wilting because leaf desiccation reduces the transpiration surface. Maximum desiccation of 100% was reached for the diploid and the fusion line 27-12 20 days after last irrigation whereas this event did not occur for the fusion lines 27-10 and 27-11. This analysis could not be conducted for the fusion line 27-09, which was the smallest line with the lowest leaf area (Table 2) and consequently consumed little water. Therefore, this line reached the target date for stopping irrigation (i.e., after 7 days at 10 vol.-% soil moisture) only 2 days before the end of the whole experimental time period, when leaves of other lines were already 100% wilted.

#### Stomatal conductance and carbohydrate concentration

**Figure 7:**
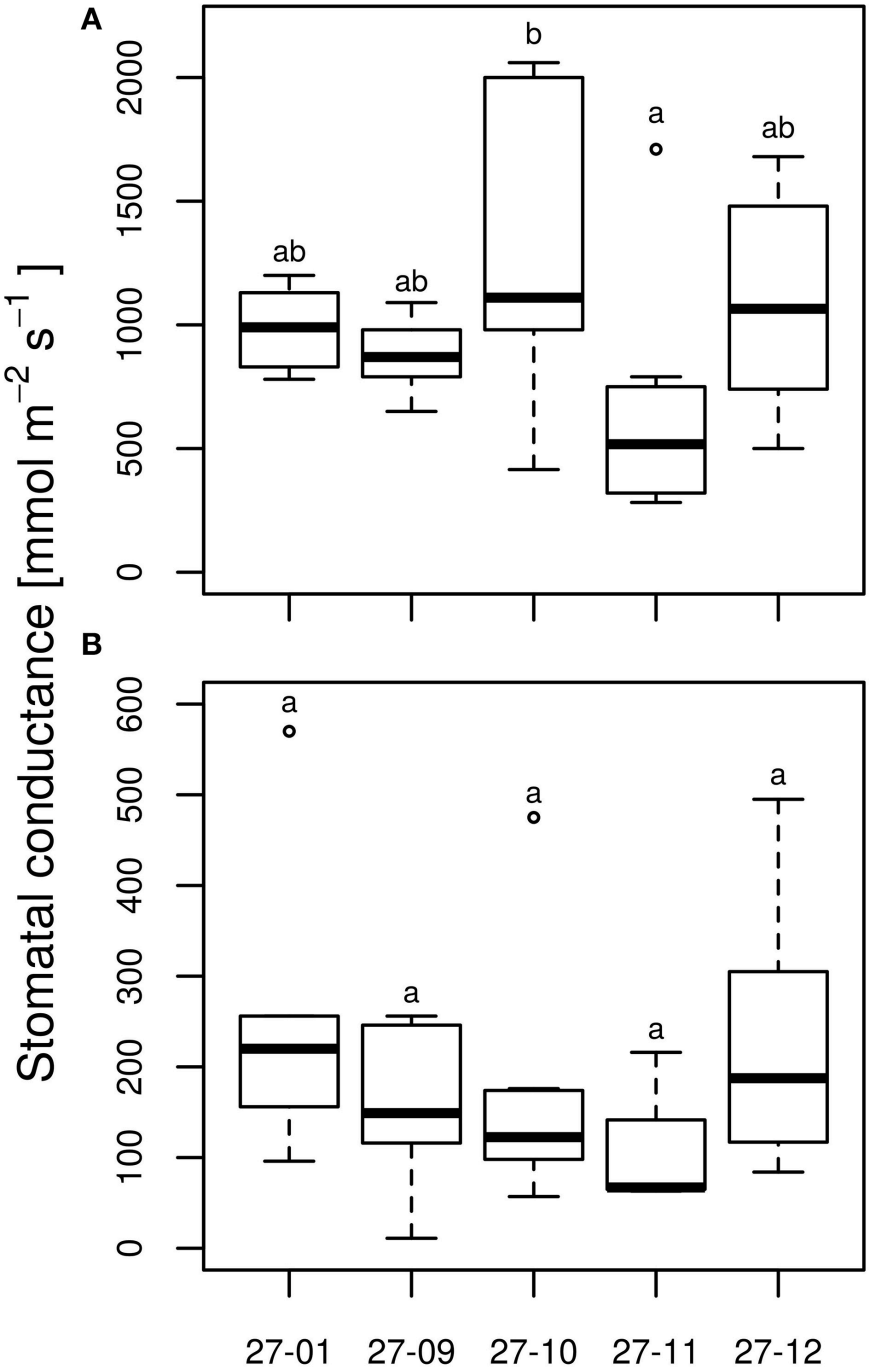
**Stomatal conductance at the end of 10 vol.-% soil moisture level (A) watered plants, (B) drought-exposed plants**. [*n* = 10 plants. Boxplots are defined as explained in Figure 3. Different letters at the top of the boxes indicate significant differences between the lines (TukeyHSD test, *p* < 0.05)].

Under well-watered conditions, no differences were observed among the stomatal conductance of the diploid line and the fusion lines (Figure 7A). All tested lines responded to dry conditions with a reduction in the stomatal conductance (Figures 7A,B) whereas this did not differ among the drought-exposed lines (Figure 7B). Here, stomatal conductance was not correlated with stomatal density (Figures 3B, 7A,B).

**Figure 8:**
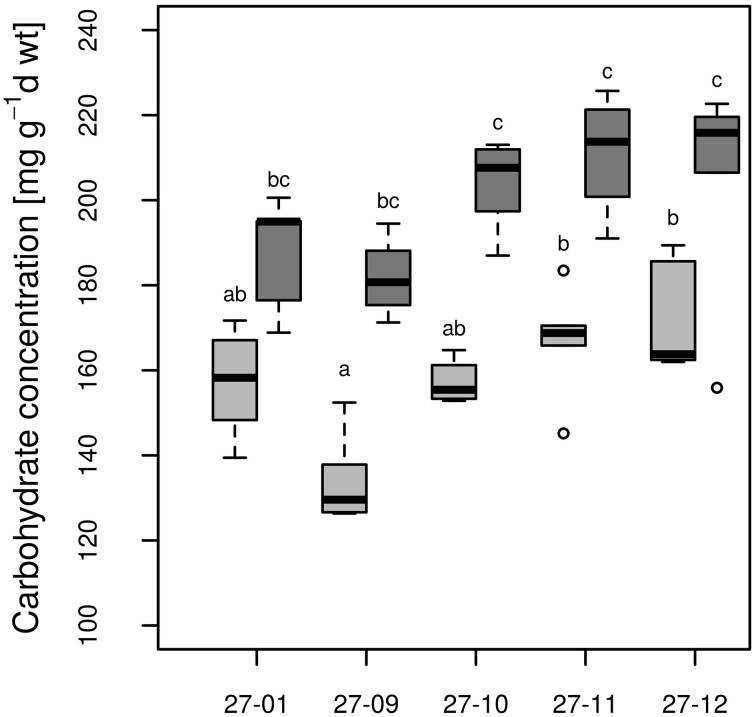
**Carbohydrate concentration at the end of 10 vol.-% soil moisture level**. [Watered plants: light gray, drought-exposed plants: dark gray, *n* = 6 plants. Boxplots are defined as explained in Figure 3. Different letters at the top of the boxes indicate significant differences between the lines (TukeyHSD test, *p* < 0.05)].

All fusion lines increased their foliar carbohydrate concentrations in response to drought (Figure 8). In the diploid line, the increase was not significant (Figure 8). Overall, the carbohydrate concentrations of the fusion lines were similar to that of the diploid line (Figure 8). Genotype and treatment interactions were not found for the parameters carbohydrate concentration and stomatal conductance (Table 3).

**Table 3:** Significance of the parameters genotype (G), treatment (T), light (L) and their interaction effects on the stomatal conductance, the carbohydrate concentration, the relative height and stem increment (^*^p < 0.05, ^**^p < 0.01, ^***^p < 0.001; ns, not significant; –, not analyzed).

**Parameter**	**G**	**T**	**L**	**G:T**	**G:L**	**L:T**
Stomatal conductance	ns	^**^	ns	ns	ns	ns
Carbohydrate concentration	^***^	^***^	–	ns	–	–
Relative height increment	^***^	^***^	–	^***^	–	–
Relative stem increment	^***^	^***^	–	^*^	–	–

#### Biomass production

**Figure 9:**
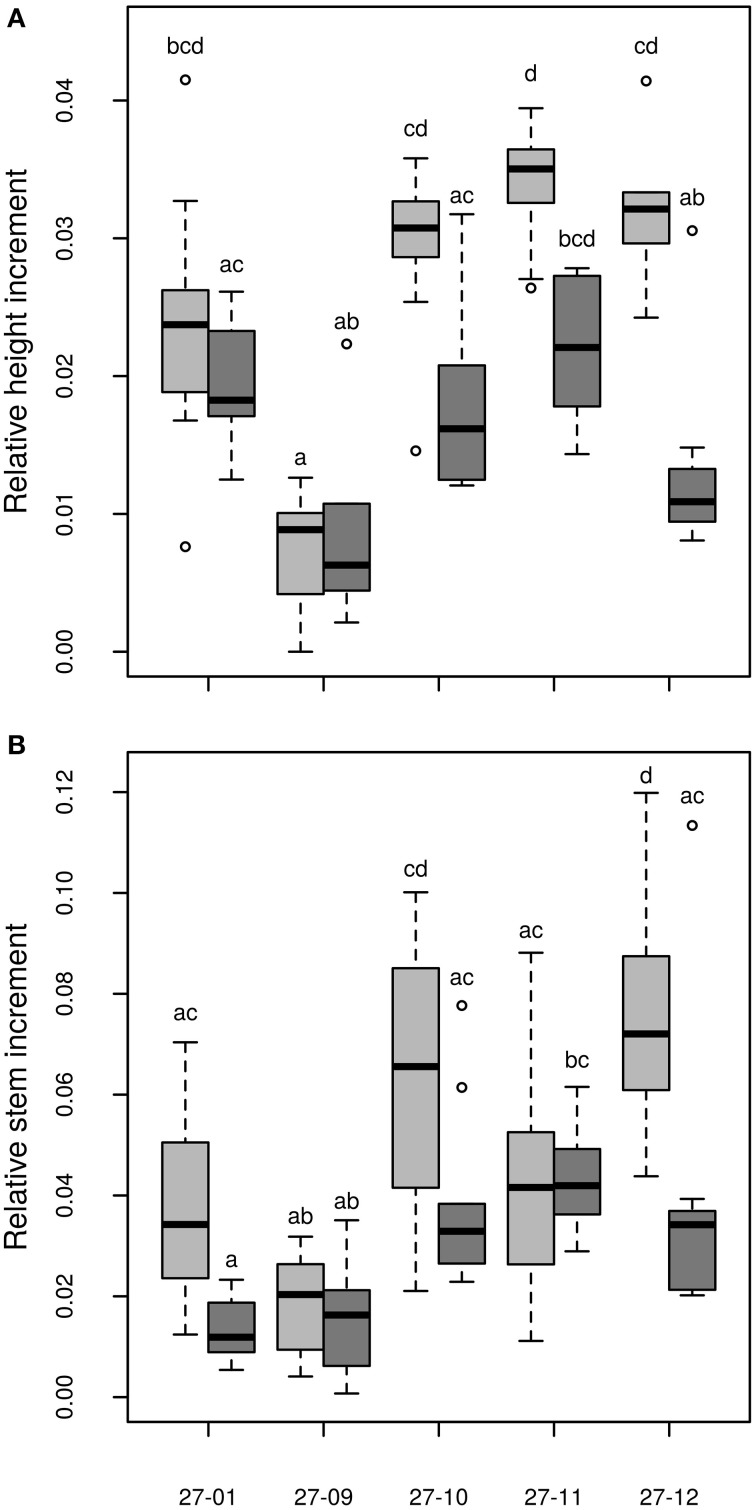
**(A)** Relative height increment, **(B)** Relative stem increment [stem without leaves, relative stem increment data are normalized to the number of days when plants were irrigated, data of the relative height increment are normalized to the number of cultured days (≡ days when plants were irrigated); watered plants: light gray, drought-exposed plants: dark gray, *n* = 10 plants. Boxplots are defined as explained in Figure 3. Different letters at the top of the boxes indicate significant differences between the lines (TukeyHSD test, *p* < 0.05)].

Among the fusion lines the relative height increment was reduced under drought in the lines 27-10, 27-11, and 27-12, but not in the line 27-09. This line probably suffered only from mild drought because this fusion line was smaller than the other lines (Figure 9A). Instead, the diploid line experienced severe drought stress but showed no reduction in relative height increment (Figure 9A). Under optimally watered conditions the relative height growth of the fusion lines 27-10, 27-11, and 27-12 was equivalent to that of the diploid line, while that of the fusion line 27-09 was significantly lower (Figure 9A). Interactions between the genotype and the treatment were detected for both relative height and stem increment (Table 3).

Dry matter of the stem is an important parameter for biomass production in short rotation coppice. We analyzed the shoot dry matter without the leaves. The relative stem increment was not decreased in the fusion lines but enhanced for the fusion line 27-12 compared to the diploid line under optimally watered conditions (Figure 9B). Under the drought treatment, relative shoot increment was equivalent to the diploid line in all fusion lines except for the fusion line 27-11 that showed an increased relative shoot increment (Figure 9B).

## Discussion

### Morphological and genetic characterization

nSSR analysis did not reveal the presence of DNA of *P. nigra* or *P. trichocarpa* × *P. deltoides* in any fusion line. Because all fusion lines were tetraploid, our results suggest that homofusions of two *P. tremula* × *P. tremuloides* protoplasts occurred.

Polyploidy often induces morphological and phenotypic variation (Liu and Wendel, 2003). A reduction in height growth, as observed here for some fusion lines, has been reported in other polyploid plants before (Porter and Weiss, 1948; Sharma and Datta, 1957; Riddle and Birchler, 2008; Deng et al., 2012). Because poplars of dry habitats were smaller than those of wet habitats (Regier et al., 2009; Yang and Miao, 2010), the observed height reduction with lower total leaf area in the tetraploid than in the diploid line might be advantageous for drought threatened habitats. We have shown here that these fusion lines also consumed less water than the diploid line.

The stomatal density is correlated with the maximum stomatal conductance in Mediterranean herbs, shrubs and woody species (Galmés et al., 2007). Decreased stomata density as observed in the fusion lines might therefore lead to enhanced drought tolerance as the stomatal conductance is reduced. However, in the fusion lines stomata lengths were enhanced. This modification of the stomatal apparatus has also been observed in other tetraploid species like *Spathiphyllum* (van Laere et al., 2010), *Platanus* (Liu et al., 2007) or *Betula* (Li et al., 1996). Hodgson et al. (2010) detected that stomatal length is positively correlated with genome size within the eudicots, *Poaceae* and other monocots without providing a causal link. The correlation of stomatal length with habitat humidity is discussed controversially (Abrams et al., 1994; Hodgson et al., 2010). For example, Hodgson et al. (2010) stated that stomatal length is correlated with habitats of high humidity. Abrams et al. (1994), in contrast, reported increasing stomatal size from wet-mesic to mesic and xeric sites in deciduous tree species. Regier et al. (2009) observed that the stomatal length was increased in one *P. nigra* genotype under water limited conditions while abaxial stomatal density was reduced. In drought tolerant tomato cultivars stomatal density was decreased, but stomatal size increased compared to drought sensitive genotypes (Kulkarni and Deshpande, 2006). These stomatal traits that have also occurred in the fusion lines might be the reason for lower water consumption as observed here. Furthermore, Ashton and Berlyn (1994) observed a reduction in the SAI from wet to xeric habitats in *Quercus* species and suggested this parameter to predict drought tolerance. More, recently QTLs for this trait have been identified and were also linked with drought tolerance (Gailing et al., 2008). Here, SAI was reduced in two fusion lines (Figure 3C) only one out of these lines consumed significantly less water related to height indicating that SAI might be a relevant parameter associated with water consumption. However, the links between this trait and DNA dosage remain obscure.

### Performance of the diploid line and the fusion lines under drought

#### Water consumption and leaf vitality

Polyploid plants often possess superior characteristics in comparison to their diploid counterparts with regard to morphological and physiological changes and their tolerance to environmental stresses (Xiong et al., 2006). In this study, we observed that all fusion lines consumed less water relative to height than the diploid line. Reduced water consumption was significant for the fusion lines 27-10 and 27-12, which both showed a total leaf area similar to the diploid line. Furthermore, there were other cases where severe height and leaf area reductions decreased water consumption of the fusion lines. The altered stomatal morphology (decreased stomatal density and increased stomatal size) and the increased leaf mass per area (a low surface to volume ratio) that is linked to dry habitats (Poorter et al., 2009) in the fusion lines 27-10 and 27-12 might have reduced the transpiration and thereby enabled the plants to use water more efficiently.

The leaf wilting was more severe for the diploid line than for the fusion lines, but two of the fusion lines showed early leaf desiccation which increased to about 50% at the end of the drought. In the genus *Populus*, leaf shedding occurs to avoid desiccation of the remaining tissue (Fischer and Polle, 2010). Blake and Tschaplinski (1992) noted that leaf shedding and the related reduction of the transpiration surface led to an increase of the water potential in the remaining tissue. In analogy to this, our finding suggests that the early-wilting fusion lines were better adapted to drought than the diploid line because they reduced the transpiration surface rapidly by desiccating a part of the leaves and thereby might have been able to delay wilting of the remaining foliage.

#### Stomatal conductance and carbohydrate concentration

The positive correlation between stomatal density and stomatal conductance as described for herbs and trees in literature (Abrams et al., 1994; Galmés et al., 2007) was not found here. Despite the variation in stomatal morphology, significant differences in the stomatal conductance between the different ploidy levels were not discovered under well-watered conditions. In polyploid *Betula* and *Lonicera* plants, stomatal conductance was less affected by drought compared to the diploid variants (Li et al., 1996, 2009), whereas the poplar fusion lines in our study exhibited the same response to drought as the diploid line.

Carbohydrates play important roles for osmotic adjustment of poplar tissues to drought stress (Danielsen and Polle, 2014). Notably, in the fusion lines the carbohydrate concentrations were more strongly increased under drought conditions than in the diploid line (Figure 8). For instance, Deng et al. (2012) also observed enhanced carbohydrate levels and increased survival of octaploid tobacco compared to its tetraploid variant under stress conditions. Accumulation of carbohydrates leads to osmotic adjustment by decreasing the osmotic potential in the cell and contributes to the stress tolerance (Touchette et al., 2009). Carbohydrates also function in osmoprotection by stabilizing proteins and membranes (Crowe et al., 1992). The stronger increase in carbohydrate concentration in the fusion lines might have enabled them to cope with dry conditions better than the diploid line because of improved osmotic adjustment and cellular protection.

#### Biomass production

Although the initial height was reduced for the fusion lines 27-09, 27-10, and 27-11 (Table 2) the relative height increment of the fusion lines 27-10, 27-11, and 27-12 was equivalent to that of the diploid line under well-watered conditions indicating no growth constraints (Figure 9A). Height growth of *Populus* is sensitive to drought at an early stage (Bogeat-Triboulot et al., 2006). McDowell et al. (2008) suggested that plants under drought conditions suffer mainly from carbon starvation because of stomatal closure. Consequently, growth is reduced. In the context of the present study, this theory implies that the fusion lines 27-10, 27-11, and 27-12 closed their stomata to reduce water loss, as a consequence their height increment decreased. Additionally, they avoided further increase in stress because they reduced the area of growing tissue that had to be supplied with water. This can also be a part of the stress avoidance response. Eventually, these measures may have led to reduced water demand, apparent as the lower water consumption of the fusion lines. In contrast, the diploid line did not respond to drought by early diminishment of height growth and thus, did not save resources. These suggestions are in line with the resource requirement hypothesis pertaining that polyploid plants grow slower and therefore, have a decreased resource demand compared to their diploid counterparts (Deng et al., 2012). In practical terms, it is obvious that in plantations for woody biomass production, genotypes are needed that represent a compromise of biomass production and drought tolerance.

In SRC stem biomass production is the most important parameter to be optimized because the woody parts are used for energy production (Karp and Shield, 2008; Polle et al., 2013). In our study, an enhancement in the relative shoot increment was detected for the fusion line 27-11 under drought and for 27-12 under control conditions, respectively compared to the diploid line. Because two of these fusion lines also showed total biomass production similar to that of the diploid line, the new genotypes appear to be reasonable alternatives for plantation in dry areas.

## Conclusion

Overall, the tetraploid lines that were generated by protoplast fusion varied significantly in morphological characteristics such as height, total leaf area and stomatal characteristics as well as in physiological traits such as carbohydrate production under drought and water consumption. The phenotypic diversity might be due to mutations in the chromosomes caused during the protoplast fusion process (Prange et al., 2012). This diversity renders the fusion lines predestined for breeding. Moreover, we could show that the fusion lines 27-10 and 27-12 desiccated a part of their foliage at an early stage of drought and were more water saving than the diploid line. All fusion lines showed a higher increase in carbohydrate concentration and a decrease in relative height increment when suffered from severe drought suggesting better drought adaptation by stress tolerance and avoidance mechanisms. Adaptability to low water supply is favorable for the SRC plantation, because it is expected that biomass plantations will be established on marginal sites with low water and mineral supply to avoid competition with agricultural land (von Wühlisch, 2012). Furthermore, a decrease in soil moisture of 5% up to 12% in the upper 10 cm deep soil is predicted for large parts of Europe in the long term (year 2080–2099) (Dai, 2012). Thus, it is likely that even production on currently moist sites will have to cope with water limitations in the future. Eventually, a sufficient supply of water is a decisive factor that determines the success of establishing SRC plantations (Helbig and Müller, 2009). Therefore, the fusion lines represent interesting candidates for the cultivation on SRC.

### Conflict of interest statement

The authors declare that the research was conducted in the absence of any commercial or financial relationships that could be construed as a potential conflict of interest.
