# Supplementary material for: Water consumption and biomass production of protoplast fusion lines of poplar hybrids under drought stress
Source: Front Plant Sci. 2015 May 19;6:330. doi: 10.3389/fpls.2015.00330 (PMC4436569; doi:10.3389/fpls.2015.00330)
Supplement: Supplementary file 1 [file Table1.PDF]

Supplementary Table 1: Results of the ANOVA analyses (Df: Degrees of freedom, Sum sq: Sums of squares, Mean Sq: Mean of squares, Pr(>F): p-value of the F statistic).

		**Df**	**Sum Sq**	**Mean Sq**	**F value**	**Pr(>F)**
One-way ANOVA						
Height						
	Genotype	4	31301	7825	28.7	< 0.001
	Residuals	45	12268	273		
Stem biomass						
	Genotype	4	3081	770.3	16.61	< 0.001
	Residuals	45	2086	46.4		
Total leaf area						
	Genotype	4	2282	570.5	8.492	< 0.001
	Residuals	19	1276	67.2		
Leaf mass per area						
	Genotype	4	31.82	7.954	19.78	< 0.001
	Residuals	611	245.68	0.402		
Stomatal length						
	Genotype	4	6324	1581.1	82.35	< 0.001
	Residuals	220	4224	19.2		
Stomatal density (adaxial leaf side)						
	Genotype	4	173812	43453	23.46	< 0.001
	Residuals	70	129663	1852		
Stomatal density (abaxial leaf side)	Genotype	4	309.9	77.48	12.25	< 0.001
	Residuals	70	442.7	6.32		
Stomatal area index						
	Genotype	4	42322483	10580621	7.959	< 0.001
	Residuals	70	93063058	1329472		
Two-way ANOVA						
Stomatal conductance						
	Genotype	4	2813404	703351	0.506	0.732
	Light	37	13394488	362013	0.261	0.999

		Df	Sum Sq	Mean Sq	F value	Pr(>F)
	Treatment	1	13412234	13412234	9.651	0.008
	Genotype:Light	28	7837321	279904	0.201	1.000
	Genotype:Treatment	4	3284655	821164	0.591	0.675
	Light:Treatment	5	2418539	483708	0.348	0.875
	Residuals	13	18065691	1389669		
Carbohydrate concentration	Genotype	4	5965	1491	7.174	< 0.001
	Treatment	1	19320	19320	92.948	< 0.001
	Genotype:Treatment	4	686	172	0.825	0.517
	Residuals	39	8106	208		
Relative height increment	Genotype	4	0.0033	$8.21e^{-4}$	10.891	< 0.001
	Treatment	1	0.0013	0.0013	17.553	< 0.001
	Genotype:Treatment	4	0.0017	$4.17e^{-4}$	5.536	< 0.001
	Residuals	85	0.0064	$7.54e^{-5}$		
Relative stem increment	Genotype	4	0.0208	0.0052	15.409	< 0.001
	Treatment	1	0.0075	0.0075	20.793	< 0.001
	Genotype:Treatment	4	0.0051	0.0013	3.518	0.010
	Residuals	87	0.0314	$3.60e^{-4}$		
