# Supplementary material for: Water consumption and biomass production of protoplast fusion lines of poplar hybrids under drought stress
Source: Front Plant Sci. 2015 May 19;6:330. doi: 10.3389/fpls.2015.00330 (PMC4436569; doi:10.3389/fpls.2015.00330)
Supplement: Supplementary file 2 [file Table2.PDF]

Supplementary Table 2: Results of the Tukey HSD test (27-01: diploid original clone, other lines: tetraploid fusion lines, diff: differences between the means, lwr: lower end point and upr: upper end point of the interval, p adj: p value after adjustment for the multiple comparisons; C: control group, D: drought treatment).

	**diff**	**lwr**	**upr**	**p adj**
Stem height				
27-09-27-01	-65.50	-86.481713	-44.518287	< 0.001
27-10-27-01	-24.80	-45.781713	-3.818287	0.013
27-11-27-01	-55.30	-76.281713	-34.318287	< 0.001
27-12-27-01	-12.25	-33.231713	8.731713	0.469
27-10-27-09	40.70	19.718287	61.681713	< 0.001
27-11-27-09	10.20	-10.781713	31.181713	0.643
27-12-27-09	53.25	32.268287	74.231713	< 0.001
27-11-27-10	-30.50	-51.481713	-9.518287	0.001
27-12-27-10	12.55	-8.431713	33.531713	0.444
27-12-27-11	43.05	22.068287	64.031713	< 0.001
Stem biomass				
27-09-27-01	-16.308	-24.960204	-7.655796	< 0.001
27-10-27-01	-1.973	-10.625204	6.679204	0.966
27-11-27-01	-13.360	-22.012204	-4.707796	< 0.001
27-12-27-01	3.819	-4.833204	12.471204	0.720
27-10-27-09	14.335	5.682796	22.987204	< 0.001
27-11-27-09	2.948	-5.704204	11.600204	0.868
27-12-27-09	20.127	11.474796	28.779204	< 0.001
27-11-27-10	-11.387	-20.039204	-2.734796	0.005
27-12-27-10	5.792	-2.860204	14.444204	0.331
27-12-27-11	17.179	8.526796	25.831204	< 0.001
Total leaf area				
27-09-27-01	-23.869559	-40.4042111	-7.3349066	0.003
27-10-27-01	-6.483065	-22.0720849	9.1059545	0.723
27-11-27-01	-15.041259	-30.6302787	0.5477607	0.062
27-12-27-01	3.815397	-11.7736229	19.4044165	0.945
27-10-27-09	17.386494	0.8518414	33.9211459	0.036
27-11-27-09	8.828300	-7.7063524	25.3629521	0.512
27-12-27-09	27.684956	11.1503034	44.2196079	< 0.001
27-12-27-10	10.298462	-5.2905577	25.8874817	0.309
27-12-27-11	18.856656	3.2676361	34.4456755	0.013
Leaf mass per area				
27-09-27-01	0.84925976	0.580283145	1.1182364	< 0.001
27-10-27-01	0.21027348	0.003207338	0.4173396	0.045
27-11-27-01	0.15047436	-0.058851208	0.3597999	0.284
27-12-27-01	0.31021406	0.111856095	0.5085720	< 0.001
27-10-27-09	-0.63898628	-0.914913965	-0.3630586	< 0.001
27-11-27-09	-0.69878540	-0.976412656	-0.4211581	< 0.001
27-12-27-09	-0.53904570	-0.808499930	-0.2695915	< 0.001
27-11-27-10	-0.05979912	-0.277984497	0.1583863	0.945
27-12-27-10	0.09994058	-0.107745596	0.3076268	0.681
27-12-27-11	0.15973970	-0.050199235	0.3696786	0.229
Stomatal length				
27-09-27-01	13.230222	10.6894694	15.7709750	< 0.001
27-10-27-01	8.664222	6.1234694	11.2049750	< 0.001
27-11-27-01	15.462222	12.9214694	18.0029750	< 0.001
27-12-27-01	9.875556	7.3348027	12.4163084	< 0.001
27-10-27-09	-4.566000	-7.1067528	-2.0252472	< 0.001
27-11-27-09	2.232000	-0.3087528	4.7727528	0.115
27-12-27-09	-3.354667	-5.8954195	-0.8139139	0.003
27-11-27-10	6.798000	4.2572472	9.3387528	< 0.001
27-12-27-10	1.211333	-1.3294195	3.7520861	0.684
27-12-27-11	-5.586667	-8.1274195	-3.0459139	< 0.001

	diff	lwr	upr	p adj
Stomatal density abaxial side				
27-09-27-01	-74.06533	-118.07116	-30.0595048	< 0.001
27-10-27-01	-97.30333	-141.30916	-53.2975048	< 0.001
27-11-27-01	-140.87067	-184.87650	-96.8648381	< 0.001
27-12-27-01	-116.18200	-160.18783	-72.1761714	< 0.001
27-10-27-09	-23.23800	-67.24383	20.7678286	0.580
27-11-27-09	-66.80533	-110.81116	-22.7995048	< 0.001
27-12-27-09	-42.11667	-86.12250	1.8891619	0.067
27-11-27-10	-43.56733	-87.57316	0.4384952	0.054
27-12-27-10	-18.87867	-62.88450	25.1271619	0.751
27-12-27-11	24.68867	-19.31716	68.6944952	0.521
Stomatal density adaxial side				
27-09-27-01	1.332268e-16	-2.571452	2.571452	1.000
27-10-27-01	2.131628e-15	-2.571452	2.571452	1.000
27-11-27-01	5.082000e+00	2.510548	7.653452	< 0.001
27-12-27-01	5.773160e-16	-2.571452	2.571452	1.000
27-10-27-09	1.998401e-15	-2.571452	2.571452	1.000
27-11-27-09	5.082000e+00	2.510548	7.653452	< 0.001
27-12-27-09	4.440892e-16	-2.571452	2.571452	1.000
27-11-27-10	5.082000e+00	2.510548	7.653452	< 0.001
27-12-27-10	-1.554312e-15	-2.571452	2.571452	1.000
27-12-27-11	-5.082000e+00	-7.653452	-2.510548	< 0.001
Stomatal area index				
27-09-27-01	316.3776	-862.5598	1495.31509	0.943
27-10-27-01	-933.5915	-2112.5290	245.34593	0.185
27-11-27-01	-1629.1829	-2808.1204	-450.24546	0.002
27-12-27-01	-1317.5329	-2496.4704	-138.59547	0.021
27-10-27-09	-1249.9692	-2428.9066	-71.03171	0.032
27-11-27-09	-1945.5605	-3124.4980	-766.62309	< 0.001
27-12-27-09	-1633.9106	-2812.8480	-454.97311	0.002
27-11-27-10	-695.5914	-1874.5288	483.34607	0.470
27-12-27-10	-383.9414	-1562.8788	794.99605	0.891
27-12-27-11	311.6500	-867.2875	1490.58744	0.946
Stomatal conductance C				
27-09-27-01	-114.0	-1228.1061	1000.1061	0.998
27-10-27-01	813.5	-300.6061	1927.6061	0.249
27-11-27-01	-364.3	-1478.4061	749.8061	0.884
27-12-27-01	96.0	-1018.1061	1210.1061	0.999
27-10-27-09	927.5	-186.6061	2041.6061	0.144
27-11-27-09	-250.3	-1364.4061	863.8061	0.968
27-12-27-09	210.0	-904.1061	1324.1061	0.983
27-11-27-10	-1177.8	-2291.9061	-63.6939	0.034
27-12-27-10	-717.5	-1831.6061	396.6061	0.370
27-12-27-11	460.3	-653.8061	1574.4061	0.766
Stomatal conductance D				
27-09-27-01	-74.40000	-222.65571	73.85571	0.608
27-10-27-01	-74.90000	-223.15571	73.35571	0.602
27-11-27-01	-117.56667	-335.79316	100.65982	0.542
27-12-27-01	-15.90000	-164.15571	132.35571	0.998
27-10-27-09	-0.50000	-148.75571	147.75571	1.000
27-11-27-09	-43.16667	-261.39316	175.05982	0.980
27-12-27-09	58.50000	-89.75571	206.75571	0.790
27-11-27-10	-42.66667	-260.89316	175.55982	0.980
27-12-27-10	59.00000	-89.25571	207.25571	0.785
27-12-27-11	101.66667	-116.55982	319.89316	0.672

	diff	lwr	upr	p adj
Carbohydrate concentration				
27-01D-27-01C	30.2280	-0.3423884	60.7983884	0.055
27-09C-27-01C	-22.3980	-52.9683884	8.1723884	0.323
27-09D-27-01C	25.0220	-5.5483884	55.5923884	0.192
27-10C-27-01C	0.5520	-30.0183884	31.1223884	1.000
27-10D-27-01C	46.4360	15.8656116	77.0063884	< 0.001
27-11C-27-01C	9.7860	-20.7843884	40.3563884	0.985
27-11D-27-01C	54.0995	21.6747066	86.5242934	< 0.001
27-12C-27-01C	15.6860	-14.8843884	46.2563884	0.778
27-12D-27-01C	47.1380	16.5676116	77.7083884	< 0.001
27-09C-27-01D	-52.6260	-83.1963884	-22.0556116	< 0.001
27-09D-27-01D	-5.2060	-35.7763884	25.3643884	1.000
27-10C-27-01D	-29.6760	-60.2463884	0.8943884	0.063
27-10D-27-01D	16.2080	-14.3623884	46.7783884	0.745
27-11C-27-01D	-20.4420	-51.0123884	10.1283884	0.447
27-11D-27-01D	23.8715	-8.5532934	56.2962934	0.316
27-12C-27-01D	-14.5420	-45.1123884	16.0283884	0.843
27-12D-27-01D	16.9100	-13.6603884	47.4803884	0.698
27-09D-27-09C	47.4200	16.8496116	77.9903884	< 0.001
27-10C-27-09C	22.9500	-7.6203884	53.5203884	0.291
27-10D-27-09C	68.8340	38.2636116	99.4043884	< 0.001
27-11C-27-09C	32.1840	1.6136116	62.7543884	0.032
27-11D-27-09C	76.4975	44.0727066	108.9222934	< 0.001
27-12C-27-09C	38.0840	7.5136116	68.6543884	0.006
27-12D-27-09C	69.5360	38.9656116	100.1063884	< 0.001
27-10C-27-09D	-24.4700	-55.0403884	6.1003884	0.216
27-10D-27-09D	21.4140	-9.1563884	51.9843884	0.383
27-11C-27-09D	-15.2360	-45.8063884	15.3343884	0.804
27-11D-27-09D	29.0775	-3.3472934	61.5022934	0.112
27-12C-27-09D	-9.3360	-39.9063884	21.2343884	0.989
27-12D-27-09D	22.1160	-8.4543884	52.6863884	0.339
27-10D-27-10C	45.8840	15.3136116	76.4543884	< 0.001
27-11C-27-10C	9.2340	-21.3363884	39.8043884	0.990
27-11D-27-10C	53.5475	21.1227066	85.9722934	< 0.001
27-12C-27-10C	15.1340	-15.4363884	45.7043884	0.810
27-12D-27-10C	46.5860	16.0156116	77.1563884	< 0.001
27-11C-27-10D	-36.6500	-67.2203884	-6.0796116	0.009
27-11D-27-10D	7.6635	-24.7612934	40.0882934	0.998
27-12C-27-10D	-30.7500	-61.3203884	-0.1796116	0.048
27-12D-27-10D	0.7020	-29.8683884	31.2723884	1.000
27-11D-27-11C	44.3135	11.8887066	76.7382934	0.002
27-12C-27-11C	5.9000	-24.6703884	36.4703884	1.000
27-12D-27-11C	37.3520	6.7816116	67.9223884	0.007
27-12C-27-11D	-38.4135	-70.8382934	-5.9887066	0.010
27-12D-27-11D	-6.9615	-39.3862934	25.4632934	0.999
27-12D-27-12C	31.4520	0.8816116	62.0223884	0.039
Relative height increment				
27-09:C-27-01:C	-0.0162724251	-0.028888059	-0.0036567909	0.003
27-10:C-27-01:C	0.0054162314	-0.007199403	0.0180318656	0.926
27-11:C-27-01:C	0.0102303859	-0.002385248	0.0228460201	0.218
27-12:C-27-01:C	0.0083019747	-0.006265305	0.0228692543	0.701
27-01:D-27-01:C	-0.0047646981	-0.017380332	0.0078509361	0.966
27-09:D-27-01:C	-0.0099540338	-0.022569668	0.0026616004	0.251
27-10:D-27-01:C	-0.0052872532	-0.018248585	0.0076740789	0.945
27-11:D-27-01:C	-0.0020197165	-0.014635351	0.0105959177	1.000
27-12:D-27-01:C	-0.0109242965	-0.023539931	0.0016913378	0.149
27-10:C-27-09:C	0.0216886564	0.009073022	0.0343042907	< 0.001
27-11:C-27-09:C	0.0265028109	0.013887177	0.0391184451	< 0.001
27-12:C-27-09:C	0.0245743998	0.010007120	0.0391416794	< 0.001
27-01:D-27-09:C	0.0115077270	-0.001107907	0.0241233612	0.105
27-09:D-27-09:C	0.0063183913	-0.006297243	0.0189340255	0.831

	diff	lwr	upr	p adj
Relative height increment				
27-10:D-27-09:C	0.0109851719	-0.001976160	0.0239465039	0.170
27-11:D-27-09:C	0.0142527086	0.001637074	0.0268683428	0.015
27-12:D-27-09:C	0.0053481286	-0.007267506	0.0179637628	0.931
27-11:C-27-10:C	0.0048141545	-0.007801480	0.0174297887	0.964
27-12:C-27-10:C	0.0028857433	-0.011681536	0.0174530229	1.000
27-01:D-27-10:C	-0.0101809294	-0.022796564	0.0024347048	0.224
27-09:D-27-10:C	-0.0153702652	-0.027985899	-0.0027546310	0.006
27-10:D-27-10:C	-0.0107034845	-0.023664817	0.0022578475	0.197
27-11:D-27-10:C	-0.0074359478	-0.020051582	0.0051796864	0.659
27-12:D-27-10:C	-0.0163405278	-0.028956162	-0.0037248936	0.002
27-12:C-27-11:C	-0.0019284112	-0.016495691	0.0126388684	1.000
27-01:D-27-11:C	-0.0149950839	-0.027610718	-0.0023794497	0.008
27-09:D-27-11:C	-0.0201844197	-0.032800054	-0.0075687855	< 0.001
27-10:D-27-11:C	-0.0155176390	-0.028478971	-0.0025563070	0.007
27-11:D-27-11:C	-0.0122501023	-0.024865737	0.0003655319	0.064
27-12:D-27-11:C	-0.0211546823	-0.033770317	-0.0085390481	< 0.001
27-01:D-27-12:C	-0.0130666728	-0.027633952	0.0015006069	0.118
27-09:D-27-12:C	-0.0182560085	-0.032823288	-0.0036887289	0.004
27-10:D-27-12:C	-0.0135892279	-0.028456895	0.0012784396	0.103
27-11:D-27-12:C	-0.0103216912	-0.024888971	0.0042455885	0.398
27-12:D-27-12:C	-0.0192262711	-0.033793551	-0.0046589915	0.002
27-09:D-27-01:D	-0.0051893357	-0.017804970	0.0074262985	0.942
27-10:D-27-01:D	-0.0005225551	-0.013483887	0.0124387769	1.000
27-11:D-27-01:D	0.0027449816	-0.009870653	0.0153606158	0.999
27-12:D-27-01:D	-0.0061595984	-0.018775233	0.0064560358	0.851
27-10:D-27-09:D	0.0046667806	-0.008294551	0.0176281127	0.975
27-11:D-27-09:D	0.0079343173	-0.004681317	0.0205499515	0.572
27-12:D-27-09:D	-0.0009702626	-0.013585897	0.0116453716	1.000
27-11:D-27-10:D	0.0032675367	-0.009693795	0.0162288687	0.998
27-12:D-27-10:D	-0.0056370433	-0.018598375	0.0073242888	0.920
27-12:D-27-11:D	-0.0089045800	-0.021520214	0.0037110542	0.404
Relative stem increment				
27-09:C-27-01:C	-0.0186664452	-0.0479112926	0.0105784022	0.551
27-10:C-27-01C	0.0262633888	-0.0013089178	0.0538356954	0.075
27-11:C-27-01:C	0.0047000611	-0.0228722455	0.0322723676	1.000
27-12:C-27-01:C	0.0376358942	0.0100635876	0.0652082008	0.001
27-01:D-27-01:C	-0.0230841388	-0.0514119910	0.0052437133	0.213
27-09:D-27-01:C	-0.0212199923	-0.0487922989	0.0063523143	0.284
27-10:D-27-01:C	0.0011058723	-0.0264664343	0.0286781789	1.000
27-11:D-27-01:C	0.0058470190	-0.0217252876	0.0334193256	1.000
27-12:D-27-01:C	0.0006388238	-0.0269334828	0.0282111304	1.000
27-10:C-27-09:C	0.0449298340	0.0156849866	0.0741746815	< 0.001
27-11:C-27-09:C	0.0233665062	-0.0058783412	0.0526113537	0.236
27-12:C-27-09:C	0.0563023394	0.0270574920	0.0855471869	< 0.001
27-01:D-27-09:C	-0.0044176936	-0.0343759349	0.0255405476	1.000
27-09:D-27-09:C	-0.0025535471	-0.0317983945	0.0266913003	1.000
27-10:D-27-09:C	0.0197723175	-0.0094725299	0.0490171649	0.468
27-11:D-27-09:C	0.0245134642	-0.0047313833	0.0537583116	0.181
27-12:D-27-09:C	0.0193052690	-0.0099395785	0.0485501164	0.503
27-11:C-27-10:C	-0.0215633278	-0.0491356344	0.0060089788	0.263
27-12:C-27-10:C	0.0113725054	-0.0161998012	0.0389448120	0.941
27-01:D-27-10:C	-0.0493475276	-0.0776753798	-0.0210196755	< 0.001
27-09:D-27-10:C	-0.0474833811	-0.0750556877	-0.0199110745	< 0.001
27-10:D-27-10:C	-0.0251575165	-0.0527298231	0.0024147901	0.104
27-11:D-27-10:C	-0.0204163698	-0.0479886764	0.0071559368	0.336
27-12:D-27-10:C	-0.0256245650	-0.0531968716	0.0019477416	0.091
27-12:C-27-11:C	0.0329358332	0.0053635266	0.0605081398	0.007
27-01:D-27-11:C	-0.0277841999	-0.0561120520	0.0005436523	0.060
27-09:D-27-11:C	-0.0259200533	-0.0534923599	0.0016522532	0.084
27-10:D-27-11:C	-0.0035941888	-0.0311664953	0.0239781178	1.000
27-11:D-27-11:C	0.0011469579	-0.0264253487	0.0287192645	1.000

	diff	lwr	upr	p adj
Relative stem increment				
27-12:D-27-11:C	-0.0040612373	-0.0316335439	0.0235110693	1.000
27-01:D-27-12:C	-0.0607200330	-0.0890478852	-0.0323921809	< 0.001
27-09:D-27-12:C	-0.0588558865	-0.0864281931	-0.0312835799	< 0.001
27-10:D-27-12:C	-0.0365300219	-0.0641023285	-0.0089577153	0.002
27-11:D-27-12:C	-0.0317888752	-0.0593611818	-0.0042165686	0.011
27-12:D-27-12:C	-0.0369970704	-0.0645693770	-0.0094247638	0.001
27-09:D-27-01:D	0.0018641465	-0.0264637056	0.0301919987	1.000
27-10:D-27-01:D	0.0241900111	-0.0041378410	0.0525178633	0.162
27-11:D-27-01:D	0.0289311578	0.0006033057	0.0572590099	0.041
27-12:D-27-01:D	0.0237229626	-0.0046048895	0.0520508147	0.182
27-10:D-27-09:D	0.0223258646	-0.0052464420	0.0498981712	0.220
27-11:D-27-09:D	0.0270670113	-0.0005052953	0.0546393179	0.059
27-12:D-27-09:D	0.0218588161	-0.0057134905	0.0494311227	0.246
27-11:D-27-10:D	0.0047411467	-0.0228311599	0.0323134533	1.000
27-12:D-27-10:D	-0.0004670485	-0.0280393551	0.0271052581	1.000
27-12:D-27-11:D	-0.0052081952	-0.0327805018	0.0223641114	1.000
