# Supplementary material for: Water consumption and biomass production of protoplast fusion lines of poplar hybrids under drought stress
Source: Front Plant Sci. 2015 May 19;6:330. doi: 10.3389/fpls.2015.00330 (PMC4436569; doi:10.3389/fpls.2015.00330)
Supplement: Supplementary file 3 [file Table3.PDF]

Supplementary Table 3: Results of the Wilcoxon signed rank test (27-01: original diploid line, other lines are fusion lines).

	**27-09**	**27-10**	**27-11**	**27-12**
Leaf wilting				
27-01	W = 38936.5, $p < 0.001$	W = 42707.5 $p < 0.001$	W = 54018.5 $p < 0.001$	W = 38581 $p = 0.009$
27-09	-	W = 12851.5 $p < 0.001$	W = 20443 $p < 0.001$	W = 12378 $p < 0.001$
27-10		-	W = 51283.5 $p < 0.001$	W = 34735 $p < 0.001$
27-11			-	W = 27442.5 $p < 0.001$
Leaf desiccation				
27-01	W = 35951 $p < 0.001$	W = 36938 $p = 0.161$	W = 51114.5 $p < 0.001$	W = 26534 $p < 0.001$
27-09	-	W = 11022.5 $p < 0.001$	W = 20771 $p < 0.001$	W = 6319.5 $p < 0.001$
27-10		-	W = 59265.5 $p < 0.001$	W = 29274.5 $p < 0.001$
27-11			-	W = 15608 $p < 0.001$
