# Supplementary material for: Water consumption and biomass production of protoplast fusion lines of poplar hybrids under drought stress
Source: Front Plant Sci. 2015 May 19;6:330. doi: 10.3389/fpls.2015.00330 (PMC4436569; doi:10.3389/fpls.2015.00330)
Supplement: Supplementary file 4 [file Table4.PDF]

Supplementary Table 4: Test statistic (exact F-Test) of the linear models for determination of the water consumption.

	Model No.	Residual sum of squares	Degrees of freedom	Sum of squares	F value	p value
Model 1: Water consumption ~ Genotype	1	581194	1	406762	100.27	< 0.001
Model 2: Water consumption ~ Genotype + Height	2	174432				
