# Supplementary material for: Water consumption and biomass production of protoplast fusion lines of poplar hybrids under drought stress
Source: Front Plant Sci. 2015 May 19;6:330. doi: 10.3389/fpls.2015.00330 (PMC4436569; doi:10.3389/fpls.2015.00330)
Supplement: Supplementary file 5 [file Table5.PDF]

Supplementary Table 5: p values of the F-Test for determining differences among the genotypes in water consumption relative to the height (the exact F-Test was applied to model 2 was used (see section 2.6). Each genotype served as reference using the R function "lm").

	**27-09**	**27-10**	**27-11**	**27-12**
**27-01**	0.527	0.002	0.063	< 0.001
**27-09**		0.043	0.195	0.019
**27-10**			0.259	0.299
**27-11**				0.067
